# Persistent polyclonal B‐cell lymphocytosis: Illustration of the great mimicker of low‐grade B‐cell lymphoma

**DOI:** 10.1002/jha2.436

**Published:** 2022-04-19

**Authors:** Dingani Nkosi, Clive S. Zent, Siba El Hussein

**Affiliations:** ^1^ Department of Pathology The University of Rochester Medical Center Rochester New York USA; ^2^ Department of Medicine The University of Rochester Medical Center Rochester New York USA; ^3^ The Wilmot Cancer Institute The University of Rochester Medical Center Rochester New York USA

1

A 40 years old woman, long‐term smoker (10 pack years), presented with 6‐month history of progressive fatigue, decreased effort tolerance and non‐deliberate weight loss. Physical examination revealed splenomegaly (24 cm on CT scan) and no lymphadenopathy. White blood cell count was 10.2 × 10^9^/L (normal range [NR] 4.0–10.0) with absolute lymphocytes of 8.7 × 10^9^ L (NR 1.2–3.7) and circulating large binucleated lymphocytes with abundant cytoplasm (Figure 1A–G). Lymphocytes were polytypic by flow cytometry analysis, and expressed CD19, CD20 and CD200 (small subset), and did not express CD5, CD10 or CD38. Serum IgM was increased at 1444 mg/dL (NR 40–230 mg/dL), with decreased IgG (384 mg/dL; (NR 700–1600) and IgA (54 mg/dL; NR 70–400). Protein electrophoresis showed no paraprotein and immunofixation electrophoresis did not show a monoclonal protein. *IgH* and *IgK* gene rearrangement studies of a blood sample by polymerase chain reaction (PCR) were negative. A bone marrow biopsy was normocellular with trilineage hematopoiesis and polytypic B‐cell lymphocytosis, comprising 50% of lymphocytes by flow cytometry analysis (Figure 1H), with binucleated lymphocytes (Figure 1I) and an immunophenotype similar to circulating lymphocytes. FISH analysis with probes for *BCL2, BCL6*, *MYC*, *TP53* and *CCND1 genes* was negative. Cytogenetic analysis of blood and bone marrow cells showed a normal female karyotype. Next‐generation sequencing for mutations occurring at high frequency in hematological malignancies and high sensitivity *MYD88* mutation analysis were negative. Infectious and malignant (hematopoietic and non‐hematopoietic) causes of lymphocytosis were ruled‐out, and the patient was diagnosed with persistent polyclonal B‐cell lymphocytosis (PPBL), a rare hematological disorder mimicking low‐grade B‐cell lymphoma with a benign clinical course. PPBL usually occurs in young to middle‐aged women who smoke, and is characterized by persistent polyclonal B‐cell lymphocytosis with binucleated lymphocytes, elevated polyclonal serum IgM and splenomegaly [1, 2]. Apart from smoking, other etiological factors associated with PPBL include chronic EBV infection and genetic predisposition [3, 4]. PPBL has also been frequently associated with chromosomal abnormalities and multiple *BCL2/IG* gene rearrangements. There is no standard treatment, but smoking cessation and splenectomy have been reported to be effective in managing patients with this condition. Awareness of this entity is key to avoid misdiagnosis and provide optimal patient care.

**FIGURE 1:**
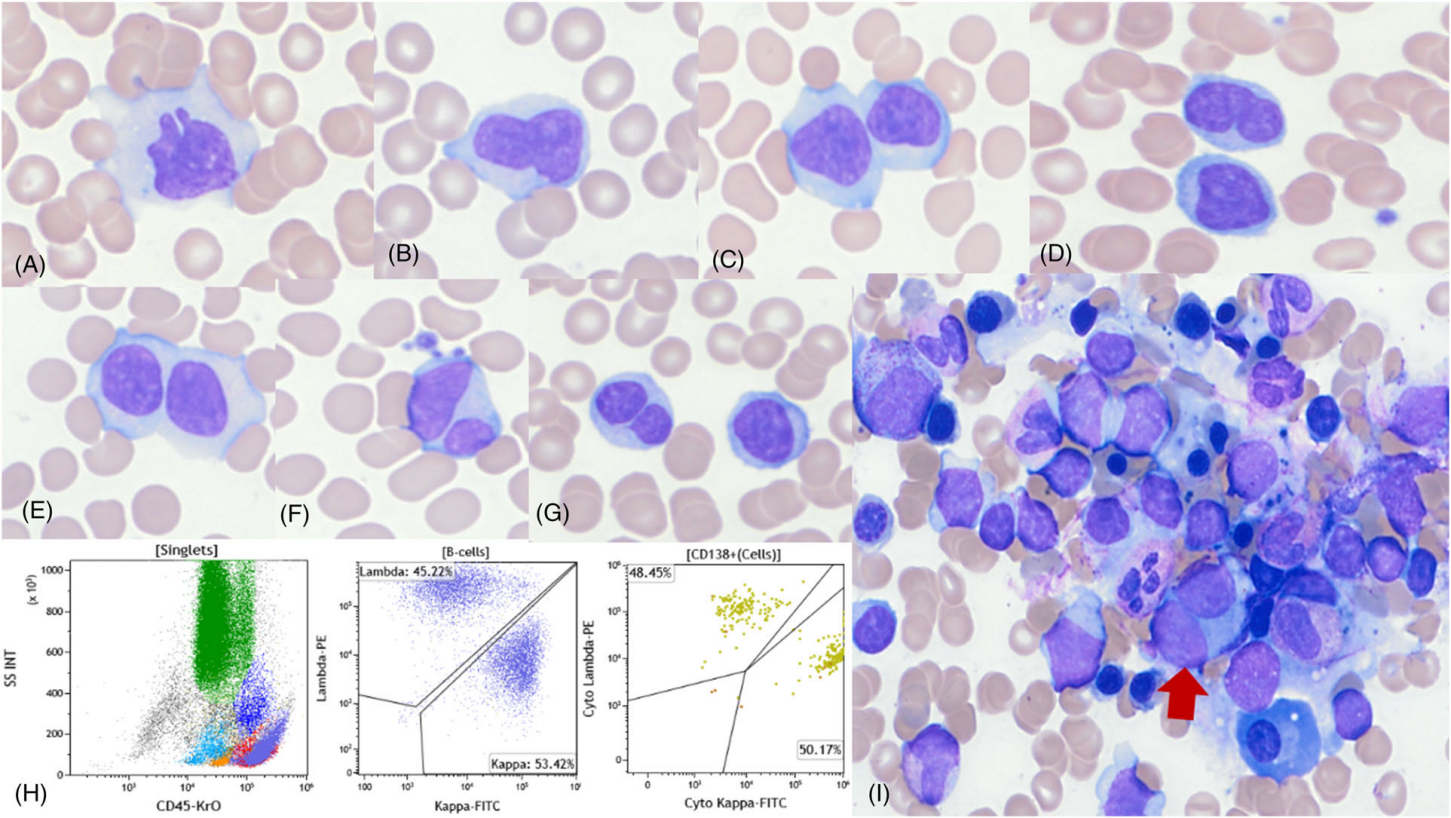
Persistent polyclonal B‐cell lymphocytosis: (A–G) Numerous circulating binucleated lymphocytes; (H) flow cytometry analysis of bone marrow aspirate shows increased B lymphocytes and plasma cells with a polytypic expression of kappa and lambda light chains and no immunophenotypic aberrancies; (I) bone marrow aspirate morphologic examination shows increased binucleated lymphocytes (red arrow), with orderly maturation of the three lineages

## CONFLICT OF INTEREST

The authors declare no conflict of interest.

## FUNDING INFORMATION

None.
